# Gas chromatography method to quantify α-monobenzoate glycerol in methanolic solutions using methyl benzoate as internal standard and a Rxi-1ms column

**DOI:** 10.1016/j.mex.2022.101910

**Published:** 2022-11-04

**Authors:** Vinícius de Macedo

**Affiliations:** Graduate Program of Chemical Engineering, Federal University of São Carlos, 13565-905, Sao Carlos, SP, Brazil

**Keywords:** 2,3-Dihydroxypropyl benzoate, Building block, Esterification, Glycerol monobenzoate

## Abstract

The 2,3-dihydroxypropyl benzoate, also known as α-monobenzoate glycerol (α-MBG), is an important building block used in the organic chemistry industry and its synthesis can be performed by the direct esterification of glycerol and benzoic acid. On the other hand, gas chromatography is a powerful laboratory tool applied for the quantification of known and unknown molar concentrations of chemical compounds. Under those conditions, I developed a method and a calibration curve for the analysis of unknown concentrations of α-MBG in methanolic solutions using a gas chromatograph equipped with a Rxi-1ms column and methyl benzoate (MBe) as an internal standard. Thus, by a suitable chromatograph setup, such as injector temperature and column oven temperature program, it was achieved a good resolution of the chromatographic peaks of each individual compound (MBe and α-MBG). Additionally, it was possible to generate an appropriated calibration curve with a corresponding equation for the calculation of unknow molar concentrations of α-monobenzoate glycerol in methanolic solutions. The highlights of the method described are:

• There were no derivatization steps in this method, only dilution of the α-monobenzoate glycerol in methanol.
• The calibration curve was made using only four calibration mixtures.
• This method also promotes the appropriate separation of glycerol, benzoic acid, and methyl benzoate, which can be useful for the analysis of the progress of an esterification reaction of glycerol and benzoic acid for the synthesis of α-MBG.

## Graphical abstract

**Figure:**
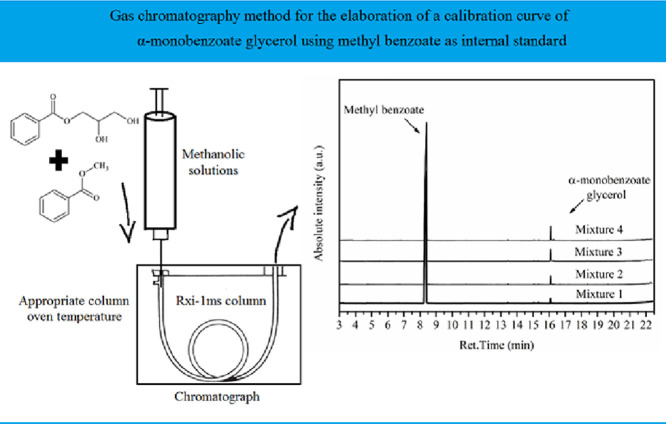

Specifications table

**Table:** 

Subject Area:	Chemistry
More specific subject area:	Analytical chemistry
Method name:	Gas chromatography method to quantify α-monobenzoate glycerol in methanolic solutions using an Rxi-1ms column
Name and reference of original method:	Adapted from “analysis of reaction products” - G. Ceni, P.C. da Silva, L. Lerin, J.V. Oliveira, G. Toniazzo, H. Treichel, E.G. Oestreicher, D. de Oliveira, Ultrasound-assisted enzymatic transesterification of methyl benzoate and glycerol to 1-glyceryl benzoate in organic solvent, Enzyme Microb. Technol. 48 (2011) 169–174. https://doi.org/10.1016/j.enzmictec.2010.10.004. [1]
Resource availability:	The α-monobenzoate glycerol was purchased from https://www.bldpharm.com/products/3376-59-8.html

## Chemicals

Methanol (>99.9%, liquid, HPLC grade), glycerol (99.5%, liquid), benzoic acid (99.5%, solid), and methyl benzoate (99%, liquid), were purchased from Sigma-Aldrich and used as received. Except the benzoic acid which was ground using an agate mortar and pestle before its use. The α-monobenzoate glycerol (1 g; 95%, pasty-like solid) was purchased from BLD Pharm China.

## Calibration mixtures preparation

Four calibration mixtures in methanol were prepared to develop a suitable method for the quantification of unknown molar concentrations of α-monobenzoate glycerol (α-MBG) using gas chromatography. The molar concentration of the components of each calibration mixture is shown in Table 1. The α-MBG was carefully weighted in a 25 mL volumetric flask and then the MBe was added as internal standard using a micropipette Labmate HTL 1000 µL. The volumetric flask was completed with methanol until meniscus was reached. The MBe was chosen as internal standard according to the literature [2,3] and the internal standard method was used as recommended for manual injections in gas chromatography [4,5]. After the preparation of the mixtures, each of them was transferred to a glass vial with PTFE lined septum for further injection into the chromatograph.

**Table 1:** Molar concentration of MBe and α-MBG in each calibration mixture.

Mixture #	methyl benzoate (mol L^−1^)	α-monobenzoate glycerol (mol L^−1^)
Mixture 1	0.1	0.0005
Mixture 2	0.1	0.00075
Mixture 3	0.1	0.001
Mixture 4	0.1	0.00125

## Chromatographic instrumentation

All the analyses were performed using a chromatograph coupled to a mass spectrometer Shimadzu model GC-QP2010Plus equipped with a flame ionization detector and a capillary column Rxi-1ms (29,8 m x 0.25 mm i.d. × 25 µm film-thickness). High purity helium was used as the carrier gas. All the mixtures were manually injected using a Hamilton glass microliter syringe model 701. Approximately 1 µL of liquid was injected into the injection port of the chromatograph each time. To avoid air bubbles in the syringe cylinder it was proceeded the careful admission of the liquid by the slow raising of the syringe piston. The identification of the start time (ST), end time (ET), retention time (RT) and peak area of the chromatograms were performed using the software GCMS Postrun GCMS Solution Version 4.45 from Shimadzu. Table 2 contains the chromatograph setup parameters and the programed column oven temperature.

**Table 2:** The chromatograph setup and the programed column oven temperature.

Chromatograph setup		Column oven temperature program		
Parameter	Set	Rate (°C min^−1^)	Final Temperature (°C)	Hold time (min)
Injector temperature	250 °С	–	40	1
Detector temperature	280°C	20	80	–
Interface temperature	150°C	10	88	–
Split ratio	1:47.8	5	100	2
Flow control	Pressure mode	20	200	4
Column pressure	76.4 kPa	20	275	–

**Fig. 1:**
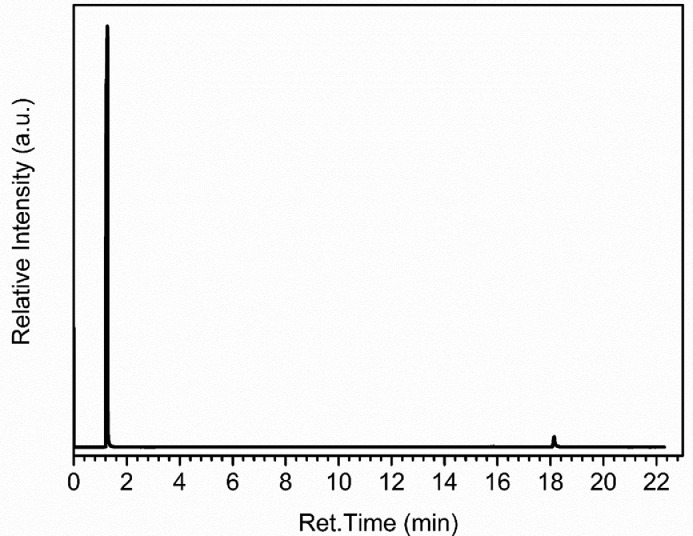
Chromatogram obtained after the manual injection of 1 µL of methanol.

Before the injection of the four calibration mixtures, it was necessary to run a blank analysis (without the injection of any type of liquid). This procedure was performed to investigate the presence of anomalous peaks that were not derived from the target molecules. Additionally, it was injected an aliquot of 1 µL of methanol into the injection port of the chromatograph to evaluate its purity and its RT. As shown in Fig. 1, the methanol RT is under 3 min, and it has high purity. However, to avoid the presence of the methanol peak in the chromatograms it was used a cutting solvent time of 3 min.

## Calibration curve

**Fig. 2:**
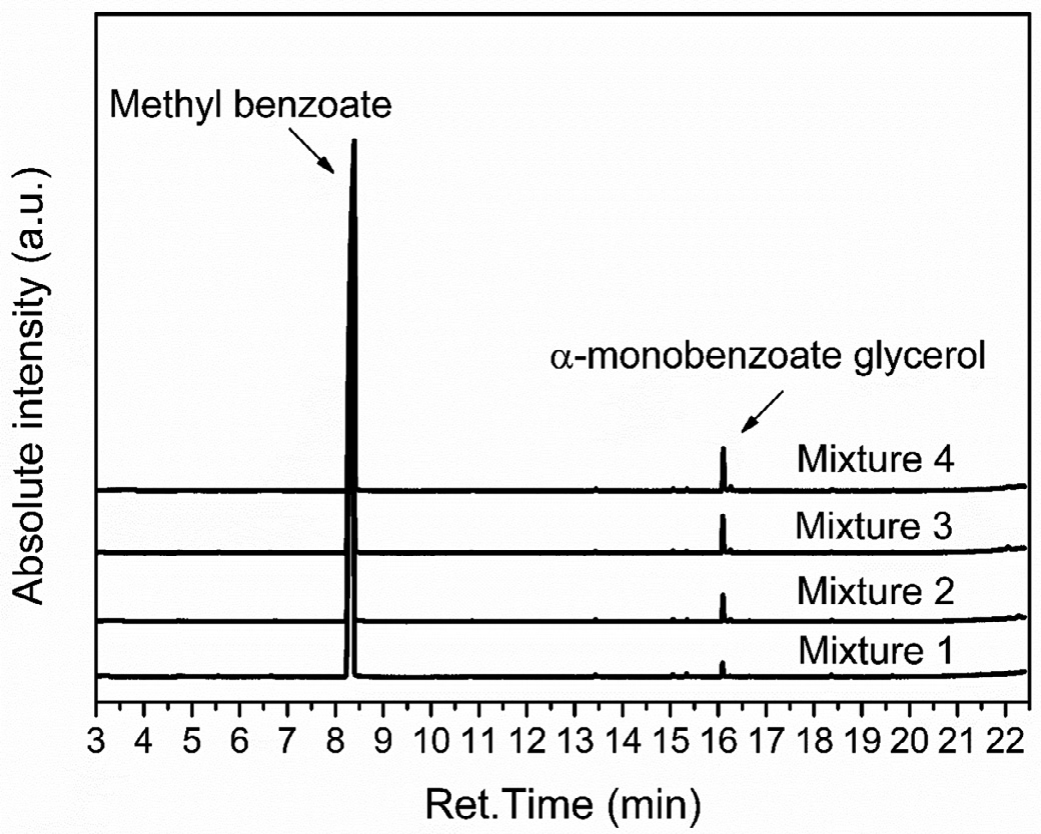
Chromatograms obtained after the injection of 1 µL of each calibration mixture containing α-MBG and MBe.

The injection of 1 µL of each calibration mixture (see Table 1) in triplicate and using the chromatograph setup (see Table 2) resulted in the chromatograms shown in Fig. 2. To avoid the overload of information, Fig. 2 shows only one of the chromatograms of each calibration mixture. As calculated by the GCMS Postrun software, the ST, ET and RT of the MBe were 8.379 ± 0.01 min, 8.386 ± 0.01 min, and 8.382 ± 0.01 min, respectively. Additionally, for the α-MBG the ST, ET and RT obtained were 16.096 ± 0.00 min, 16.102 ± 0.01 min, and 16.099 ± 0.01 min, respectively.

Table 3 shows the integrated peak area of each individual peak of α-MBG (*Area_α-MBG_*) and MBe (*Area_MBe_)* and the integrated peak area ratio of α-MBG and MBe (Area_α-MBG_/Area_MBe_). These data were obtained from the chromatograms of each calibration mixture exhibited in Fig. 2 and all the peak integration was performed using the GCMS Postrun software.

**Table 3:** Integrated peak area of the α-MBG and the MBe and the integrated peak area ratio of α-MBG and MBe.

	*Mixture #1*			*Mixture #2*		
*Injection #*	*1*	*2*	*3*	*1*	*2*	*3*
*Area_MBe_*	*14234440*	*15301030*	*16245386*	*16344923*	*15369650*	*14425339*
*Area_α-MBG_*	*192212*	*191124*	*169097*	*330498*	*339636*	*324287*
*Area_α-MBG_/Area_MBe_*	*0.014*	*0.012*	*0.010*	*0.020*	*0.022*	*0.022*

	*Mixture #3*			*Mixture #4*		
*Injection #*	*1*	*2*	*3*	*1*	*2*	*3*
*Area_MBe_*	*16819476*	*16721501*	*16685496*	*16252861*	*16261312*	*16053204*
*Area_α-MBG_*	*460781*	*468322*	*477846*	*510886*	*598407*	*535983*
*Area_α-MBG_/Area_MBe_*	*0.027*	*0.028*	*0.029*	*0.031*	*0.037*	*0.033*

**Fig. 3:**
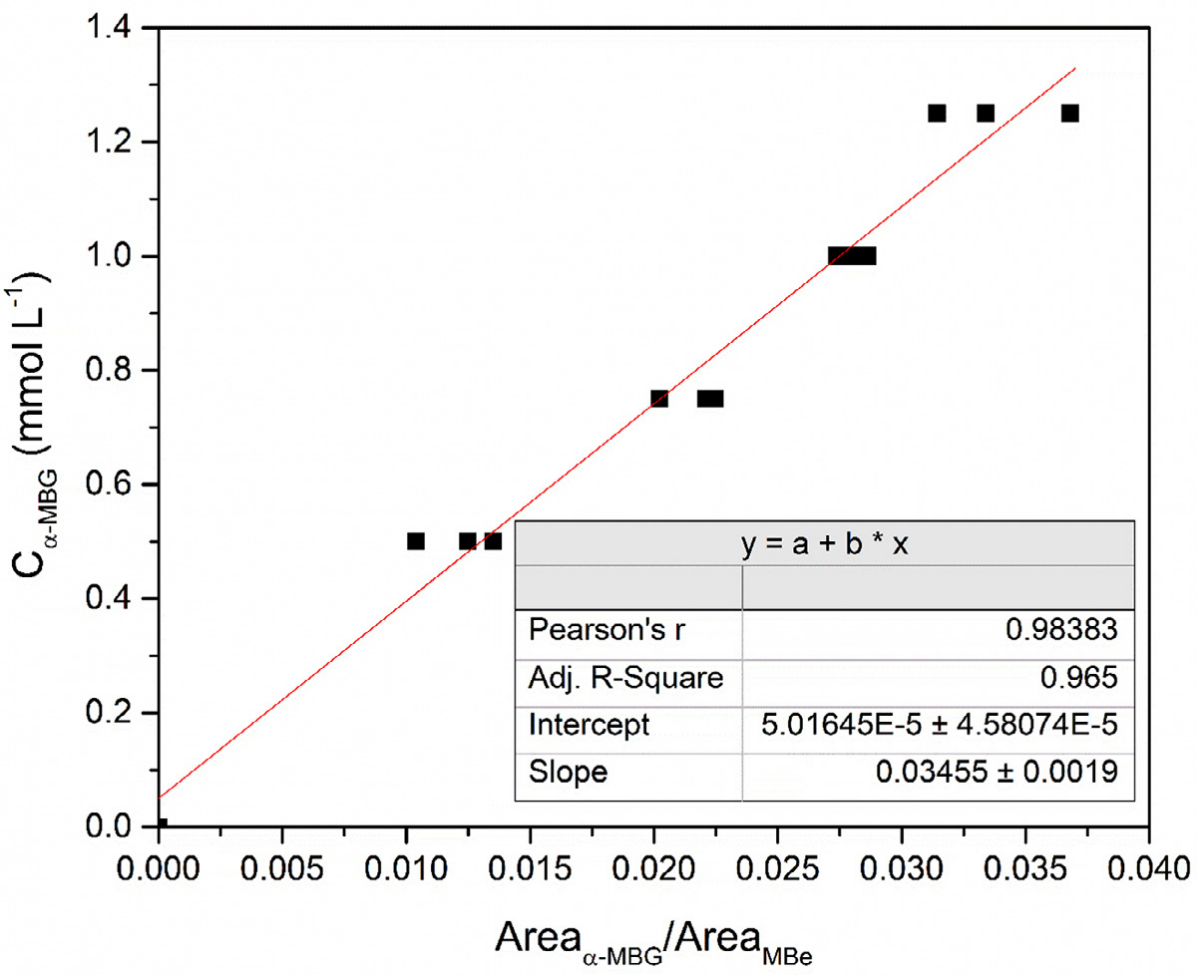
Calibration plot using the internal standard method. Y-axis are composed of the α-MBG concentration while the X-axis are composed of the integrated peak area ratio of α-MBG and MBe. Each calibration mixture had its injection performed in triplicate.

Fig. 3 shows the calibration plot and the calibration curve obtained using the data exhibited in Table 3. The only two parameters required were the α-MBG molar concentration (C_α-MBG_) in the calibration mixture (see Table 1) and the Area_α-MBG_/Area_MBe_ (see Table 3). The calibration curve was adjusted using the linear fit available in the software Origin PRO. The software yields a straight line and gives its intercept and slope based in a linear equation.

Based on the slope and intercept shown in Fig. 3, Eq. 1 was formulated. This equation allows the calculation of an unknown molar concentration of α-MBG in a methanolic solution in function of the peak area ratio of α-MBG and MBe.

(1) $$C_{\alpha -MBG}=0,03455*\frac{Area_{\alpha -MBG}}{Area_{MBe}}+5,01645\;x\;10^{-5}$$

**Fig. 4:**
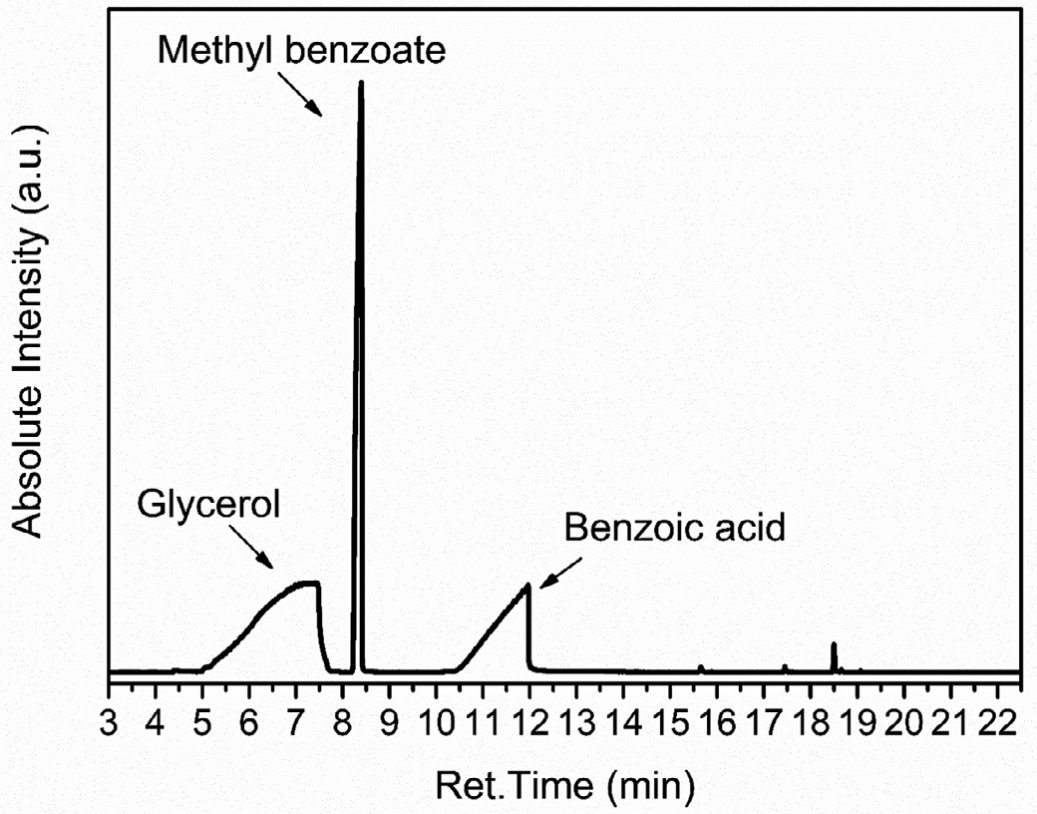
Chromatogram obtained after the injection of 1 µL of a methanolic solution containing glycerol, benzoic acid, and methyl benzoate.

The solventless esterification of glycerol and benzoic acid is one of the reactions paths that the α-MBG is synthesized [2,^3^,^6^,7]. While performing this specific esterification reaction, a researcher could have a methanolic solution containing glycerol, benzoic acid, α-MBG and MBe to analyze by gas chromatography using a chromatograph equipped with a Rxi-1ms column. Thus, to understand if this method is suitable for the analysis of this solution, a similar mixture containing glycerol, benzoic acid and MBe was made and 1 µL of it was injected in the chromatograph. The chromatogram obtained using the chromatograph setup exhibited in Table 2 is shown in Fig. 4.

According to Fig. 4, the developed method is suitable for the separation of the components of a mixture containing glycerol, benzoic acid, and methyl benzoate. Given a hypothetical situation, in the case of the analysis of the progress of an esterification reaction between glycerol and benzoic acid, if the α-MBG is formed, its peak should elute at approximately 16 min. In this way, no peak overlap is expected. Additionally, in some chromatograms, a slight overlap was noticed at approximately the ET of the glycerol peak and the ST of the MBe peak. To overcome this, the mixture in the vial should be re-injected, shaken, or diluted before proceeding to another injection. This should increase the resolution of the peaks.

## Declaration of Competing Interest

The author declare that he has no known competing financial interests or personal relationships that could have appeared to influence the work reported in this paper.

## Data Availability

Data will be made available on request.
